# Protein-tyrosine phosphorylation interaction network in *Bacillus subtilis* reveals new substrates, kinase activators and kinase cross-talk

**DOI:** 10.3389/fmicb.2014.00538

**Published:** 2014-10-22

**Authors:** Lei Shi, Nathalie Pigeonneau, Magali Ventroux, Abderahmane Derouiche, Vladimir Bidnenko, Ivan Mijakovic, Marie-Françoise Noirot-Gros

**Affiliations:** ^1^Institut National de la Recherche Agronomique, UMR1319 MicalisJouy-en-Josas, France; ^2^Systems and Synthetic Biology, Department of Chemical and Biological Engineering, Chalmers University of TechnologyGothenburg, Sweden

**Keywords:** protein phosphorylation, protein-protein network, bacterial protein kinase, bacterial cell division, bacterial signaling

## Abstract

Signal transduction in eukaryotes is generally transmitted through phosphorylation cascades that involve a complex interplay of transmembrane receptors, protein kinases, phosphatases and their targets. Our previous work indicated that bacterial protein-tyrosine kinases and phosphatases may exhibit similar properties, since they act on many different substrates. To capture the complexity of this phosphorylation-based network, we performed a comprehensive interactome study focused on the protein-tyrosine kinases and phosphatases in the model bacterium *Bacillus subtilis*. The resulting network identified many potential new substrates of kinases and phosphatases, some of which were experimentally validated. Our study highlighted the role of tyrosine and serine/threonine kinases and phosphatases in DNA metabolism, transcriptional control and cell division. This interaction network reveals significant crosstalk among different classes of kinases. We found that tyrosine kinases can bind to several modulators, transmembrane or cytosolic, consistent with a branching of signaling pathways. Most particularly, we found that the division site regulator MinD can form a complex with the tyrosine kinase PtkA and modulate its activity *in vitro*. *In vivo*, it acts as a scaffold protein which anchors the kinase at the cell pole. This network highlighted a role of tyrosine phosphorylation in the spatial regulation of the Z-ring during cytokinesis.

## Introduction

In eukarya, protein phosphorylation on serine/threonine and tyrosine residues is catalyzed by the Hanks-type family of protein kinases (Hanks et al., 1988). In bacteria, Hanks-type kinases catalyze phosphorylation of proteins only on serine and threonine residues (Pereira et al., 2011), and a distinct family of kinases, termed bacterial tyrosine kinases (BY-kinases), phosphorylate proteins on tyrosine (Grangeasse et al., 2007). BY-kinases have been initially described as autophosphorylating enzymes involved in exopolysaccharide synthesis and export (Whitfield, 2006). More recently, it became apparent that they phosphorylate many protein substrates and regulate their activity (Shi et al., 2010). Arguably the best characterized bacterial system with respect to physiological substrates of BY-kinases is the Firmicute model organism *Bacillus subtilis* (Mijakovic et al., 2005b). *B. subtilis* possesses a well characterized BY-kinase PtkA (Mijakovic et al., 2003) and a putative BY-kinase PtkB (EpsB). In order to phosphorylate its substrates, PtkA requires a transmembrane activator TkmA (Mijakovic et al., 2003), which is encoded by the same operon as the kinase. PtkB-encoding operon also codes for a transmembrane activator termed TkmB (Mijakovic et al., 2005b). While structurally the TkmB/PtkB pair constitutes a *bona fide* BY-kinase, its activity toward substrates has never been experimentally demonstrated. Recent study suggested that TkmB interaction with PtkB could modulate its function in biofilm formation in *B. subtilis* (Gerwig et al., 2014). Homologs of TkmA and TkmB exist also in proteobacteria, but there they are “fused” with the BY-kinases in a single polypeptide chain, encoded by a single gene (Jadeau et al., 2012). The rationale behind these different architectures has puzzled researchers for years. One hypothesis suggests that dissociation of BY-kinases from transmembrane activators in Firmicutes may enable these kinases to interact with alternative (possibly cytosolic) modulator proteins (Shi et al., 2010).

*B. subtilis* PtkA is known to phosphorylate a broad spectrum of substrates, including UDP-glucose dehydrogenases (Mijakovic et al., 2003), single-stranded DNA-binding proteins (Mijakovic et al., 2006), transcription factors (Derouiche et al., 2013) and other enzymes (Jers et al., 2010). Cells devoid of PtkA exhibit a pleiotropic phenotype with defects in cell cycle (Petranovic et al., 2007) and biofilm formation (Kiley and Stanley-Wall, 2010). In addition to regulating the enzyme activity of certain substrates, PtkA also seems to affect sub-cellular localization of others (Jers et al., 2010). Bacterial Hanks-type serine/threonine (Ser/Thr) kinases are equally promiscuous toward their substrates (Pereira et al., 2011). This is exemplified by the Hanks-type kinase PrkC from *B. subtilis*, which phosphorylates the essential translation factor EF-G (Gaidenko et al., 2002) and the enzymes involved in carbohydrate metabolism: the transaldolase YwjH, the glutamine synthetase GlnA, the isocitrate dehydrogenase Icd and the acetolactate-decarboxylase AlsD (Pietack et al., 2010). While recent phosphoproteomic studies in *B. subtilis* have revealed about a dozen proteins phosphorylated on tyrosine, and many more on serine and threonine (Macek et al., 2007; Soufi et al., 2010), identification of kinases responsible for these phosphorylation events *in vivo* remains a major challenge. The full complement of protein kinases in *B. subtilis* includes BY-kinases PtkA and PtkB (Mijakovic et al., 2003), the serine/threonine kinases of the Hanks type PrkC, PrkD, and YabT (Madec et al., 2002; Bidnenko et al., 2013), the unique serine/threonine kinase HprK/P (Galinier et al., 1998), the two-component-like serine/threonine kinases RsbT, RsbW, and SpoIIAB (Garsin et al., 1998; Pane-Farre et al., 2005), and 37 standard two component system kinases (Fabret et al., 1999; Kobayashi et al., 2001). The complement of corresponding phosphatases is much smaller, including the phosphotyrosine-protein phosphatases PtpZ (YwqE), YfkJ, and YwlE (Mijakovic et al., 2005a; Musumeci et al., 2005), and phosphoserine/threonine-protein phosphatases PrpC and SpoIIE (Duncan et al., 1995; Obuchowski et al., 2000). In addition, an arginine phosphorylation system has been described recently in *B. subtilis*, involving the kinase McsB and the phosphatase YwlE (Elsholz et al., 2012).

To address these crucial questions on the physiological relationships of the kinases, their substrates and alternative kinase modulators, we set out to construct a high confidence protein-protein interaction network centered on known BY-kinases, activators and phosphatases in *B. subtilis*. Our yeast two-hybrid interactome approach identified numerous candidates of high biological relevance for interactions with protein kinases and cognate phosphatases. In addition, it detected clustering of shared substrates at the interface between kinase and phosphatase nodes. It confirmed several known kinase-substrate relationships, and revealed a number of new ones, notably substrates involved in DNA replication, transcriptional regulation and cell division. Most importantly, we identified the division site selection factor MinD as an anchoring protein which modulates PtkA activity. Our approach also pointed to the existence of cross-talk between the BY-kinase and the Hanks-type Ser/Thr kinase interaction networks, revealing that the two are tightly interconnected. This suggests that bacterial protein kinases (BY- and Ser/Thr Hanks-type) could be regulated by complex signaling cascades, similar to the ones found so far only in eukarya. This study constitutes the most comprehensive view of a bacterial signaling network based on protein phosphorylation to date.

## Material and methods

### Construction of vectors for protein expression

All PCR amplifications were performed using *B. subtilis* 168 genomic DNA as template and primers as listed in Table S4. To construct the knock-out *minD* mutant, a PCR-amplified internal fragment of the *minD* gene was inserted into pMUTIN2 (Vagner et al., 1998). To obtain the CFP-N terminally fused proteins, *minD* and *ptkA* gene coding sequences were first inserted into a pSG1911 plasmid derivative (Feucht and Lewis, 2001) prior to insertion at the *amyE* platform locus. For protein expression and purification, PCR fragments were inserted into plasmid pQE-30 Xa (Qiagen) to get the 6xHis-tag fusion proteins. Strep-tagged versions of proteins were obtained from a pQE-30 vector with His6-tag replaced by the strep-tag (Jers et al., 2010). For yeast two hybrid the PCR-amplified gene coding sequences (listed in Table S4-A) were cloned into the bait vector pGBDU-C1, in frame with the binding domain of Gal4 (James et al., 1996), and transformed in PJ69-4A yeast haploid strain (James et al., 1996).

### Strain constructions

Stains used in this study as well as primers employed for constructions are precised in Table S4-A,B. The *zapA::Spc*^R^ *disruption* mutation was transferred into Δ*ptkA* background by transformation of the competent Δ*ptkA* cells with chromosomal DNA from LH165 (L. Hamoen; Kawai and Ogasawara, 2006) to spectinomycin resistance. The *minD* gene was inactivated using the vector pMUTIN2 (Vagner et al., 1998). *MinD* inactivation mutation was then transferred into the Δ*ptkA* background by transformation with pMUTIN::*minD* to erythromycin resistance.

### Yeast two hybrid screenings of a *B. subtilis* genomic library

The bait vectors were used to screen the pGAD-expressed *B. subtilis* genomic library in yeast using a previously described mating strategy (Noirot-Gros et al., 2002; Marchadier et al., 2011). Colonies were then tested for their ability to grow on SC –LUH and -LUA plates (lacking leucine, uracil, histidine or adenine). The prey candidates were identified by PCR amplification and sequencing of the DNA inserts from the pGAD plasmids. The potential false-positive interactions were eliminated experimentally using a specificity assays as previously described (Noirot-Gros et al., 2002). About 3–5 × 10^7^ yeast haploid cells expressing the BD-bait protein were mated against 3 x10^8^ haploid cells of complementary mating type, expressing the *B. subtilis* genomic library of AD-fusion prey protein fragments (Noirot-Gros et al., 2002). Mating efficiencies (15–30%) allowed to test up to 10^8^ possible interactions in one screen. For each screen, the mixture of mated cells was plated on rich medium and incubated for 4–5 h at 30°C, then collected, washed and spread on selective SC-LUH (synthetic complete medium lacking leucine, uracil, and histidine) plates supplemented with 0.5 mM 3-aminotriazole. After 7–10 days at 30°C, the His+ colonies were transferred on SC-LUA (synthetic complete medium lacking leucine, uracil, and adenine) plates for 3 days at 30°C. All the His+ Ade+ colonies were subjected to PCR amplification targeting AD prey-inserts were sequenced and compared with the *B. subtilis* genome.

### Interaction specificity assay of identified bait-preys protein pairs

False-positive interactions were experimentally removed by a specificity assay. Main sources of false positives in yeast two hybrid are (i) auto-activation of the reporter genes independently to the ability of the targeted protein to bind to a protein partner, (ii) proteins with stickiness properties and (iii) modification of the basal reporter gene expression in the yeast diploid cell. This last category mostly gives non reproducible growth phenotypes. False positive were eliminated experimentally by rescuing the protein-encoding prey plasmids from the His+ Ade+ colonies and reintroducing in PJ69–4α strain in order to perform binary interaction test assays. The rescue of the prey plasmid from diploid cells was carried out using a “gap-repair” cloning procedure as described elsewhere (Weir and Keeney, 2014). Using oligonucleotides priming upstream and downstream of the DNA insert, the prey gene fusion flanked by 150 pb homologous to the vector was amplified by PCR and co-transformed with pGAD linearized vector (digested with appropriated restriction sites within the MCS), in fresh yeast haploid cells. The yeast cell expressing the recombination-based reassembled prey plasmid were then mated against compatible haploid cells carrying (i) an empty pGBDU vector expressing and infused Gal4-BD domain, (ii) a vector expressing the targeted BD-bait protein and (iii) expressing more than three unrelated BD-protein fusions. An interaction is qualified as specific when reproduced twice with the original bait protein, without exhibiting auto-activation nor any unspecific stickiness phenotypes. As already observed (Noirot-Gros et al., 2002) 100% of the prey candidates identified as overlapping fragments of a given gene (class C1, Table S1) were found to be specific while only 20% of the prey proteins found as an unique fragment (class C2, Table S1) have passed the specificity test. In total, a given protein pair qualified as “specific” would have trigger interacting phenotypes up to three time (once during the genomic screen and at least twice during the matricial specificity test assay). The resulting network of specific bait-prey interactions is thus strongly enriched in complex with biological relevance. The protein interactions from this publication have been submitted to the IMEx consortium through IntAct (http://www.imexconsortium.org) (Orchard et al., 2014) and assigned the identifier IM-22270.

### PPI network construction

In the network the specific protein pairs were represented as nodes connected with edges using the open source software tool Cytoscape 3.0. The network was built from data on connected bait and preys as listed in Table S2.

### Proteins expression and purification

*E. coli* K12 NM522 and M15 (expressing chaperonin GroEL/GroES) were used for vector construction and protein purification, respectively. Cells were routinely grown in LB medium supplemented with appropriated antibiotics. Protein synthesis and purifications were carried out as described previously (Mijakovic et al., 2003). Induction of expression was initiated at OD_600_ = 0.6 by the addition of IPTG (1 mM). Cells were harvested 3 h later and sonicated. From crude extracts, the 6xHis- or Strep-tagged proteins were purified using Ni-NTA (Qiagen), or Strep-Tactin affinity chromatography (Novagen), prior to desalting by PD-10 columns (GE Healthcare). Protein purity was estimated by scanning densitometry of coomassie stained SDS-Page gels (Table S5).

### Far-western blotting

Proteins were separated by 12% SDS-PAGE and transferred onto nitrocellulose membranes. Proteins were then renatured (Wu et al., 2007) and incubated with MinD labeled with Strep-Tactin (conjugated to horse radish peroxidase, IBA) (Machida and Mayer, 2009) in TBS-T buffer (50 mM Tris, pH 7.5, 150 mM NaCl, 0.05% Tween 20) containing 1% BSA. For labeling, 50 μl of 0.1 μg/μl Strep-tagged MinD was incubated with 5 μ g Strep-Tactin on ice for 1 h. Signal was detected by AEC staining Kit (Sigma).

### *In vitro* phosphorylation assays

Proteins were incubated at 37°C in a reacting buffer (50 mM TrisHCl pH 7.5, 100 mM NaCl, 5 mM MgCl_2_, 5% glycerol) containing 50 μM ATP and 20 μCi/mmol [γ−^32^P]-ATP, for 1 h, prior to migration on an 8–12% SDS-polyacrylamide gel. The radioactive signal was captured by a FUJI phosphoimager (Mijakovic et al., 2003). *In vitro* phosphorylation of RecA by PtkA was performed by mixing PtkA (2.5 μM) and RecA (5 μM) in the absence or presence of TkmA (2.5 μM). Phosphorylation and dephosphorylation of RecA by YabT and SpoIIE, was performed by incubating RecA (1 μM) with YabT (0.5 μM). Then SpoIIE (5 μM) was added to the reaction. Phosphorylation and dephosphorylation of RacA by YabT and SpoIIE was performed by mixing RacA (0.8 μM) with YabT (0.15 μM) at 37°C for 1 h. Centricon was used to remove ATP from the sample, prior to addition of SpoIIE (2 μM) and DnaK (0.8 μM). Phosphorylation of DnaC by PrkD was performed by mixing 1 μM of DnaC/I stable co-purified complex with 0.1 μM PrkD. Only DnaC (50.6 kD vs. 30.1 kD for DnaI) was found to be phosphorylated by PrkD. MinD potential kinase activity was assayed by mixing equimolar concentrations of TkmA, PtkA K59M, and MinD (2 μM). *In vitro* phosphorylation of DivIVA was assayed by mixing equimolar concentrations of PtkA and TkmA (2 μM) with DivIVA (3.5 μM). MinD-mediated activation of PtkA was tested by incubating PtkA (2 μM) with 0.5, 1.0, 2.0, 4.0, and 8.0 μM of MinD. All the *in vitro* phosphorylation assays were reproduced at least three time.

### Fluorescent microscopy

Strains were grown in liquid LB medium containing appropriated antibiotic overnight at 37°C prior to 1/1000 dilution in LB. Cell were then grown up to OD_600_ 0.3. Samples were mounted on 1.2% agarose pads for examination of living cells by epifluorescent microscopy. The plasma membrane of *B. subtilis* cells were labeled using the vital stain FM 4–64 and nucleoids were stained with DAPI. CFP/GFP fluorescence was observed using appropriated dichroic filter sets.

## Results

### A protein-protein interaction network centered on BY-kinases

A network of high biological significance was constructed using a yeast two-hybrid interactome walking approach, comprising a strategy for systematic elimination of false positives (Noirot-Gros et al., 2002; Marchadier et al., 2011). We first performed genome-wide yeast two-hybrid screens of a *B. subtilis* library to identify the proteins that physically associated with the BY-kinases PtkA and PtkB, their cognate modulators TkmA and TkmB, and the three identified phosphotyrosine-protein phosphatases PtpZ (Mijakovic et al., 2005a), YwlE and YfkJ (Musumeci et al., 2005). To increase the likelihood of identifying potential kinase substrates, we also used PtkA mutant derivatives as baits, carrying point mutations either within the catalytic pocket (D81A or K59M) or within the C-terminal cluster with autophosphorylation sites (Y225F, Y227F, and Y228F) (Mijakovic et al., 2003).

In addition to the expected interactions between the BY-kinases and their respective modulators PtkA-TkmA and PtkB-TkmB, we observed a cross-connection between PtkB and TkmA as well as between TkmA and TkmB (Figure S1). The phosphotyrosine-protein phosphatase PtpZ appeared to interact with TkmA, whereas YwlE and YfkJ, the two low molecular weight phosphotyrosine-protein phosphatases (LMPTPs), lay separately from the BY-kinase and modulators (Figure S1). These findings are in agreement with previous studies indicating that PtpZ is the phosphatase dedicated to dephosphorylating substrates of PtkA/TkmA (Mijakovic et al., 2003, 2005a, 2006). *B. subtilis* LMPTPs presumably have a different role in stress resistance (Musumeci et al., 2005). Furthermore, we noticed that PtpZ shares common interactants with PtkB, suggesting that PtpZ might also act on putative substrates of PtkB (Figure S1). Common prey proteins were also found between the phosphatases YwlE and YfkJ, indicating they may have redundant activities. The BY-kinase PtkA also interacted with SalA and MinD, two MRP-like/MinD-family ATPases that bear structural resemblance with BY-kinases (Mijakovic et al., 2005b). Additionally, MinD was found to interact with PtkB and PtpZ. Last but not least, this first round of screens also revealed an interaction between the Ser/Thr kinase of the Hanks-type, YabT, and TkmA, the activator of the BY-kinase PtkA.

We then performed a second round of genomic screenings using MinD, SalA, and YabT as baits (Figure S1). In addition, we completed this set with the YabT-cognate phosphoserine-protein phosphatase SpoIIE. Interestingly, SalA and MinD were found to connect with Hanks-type Ser/Thr kinases. Indeed, save for its known interaction with the cell division regulator MinC, MinD also made contact with YabT while SalA interacted with PrkD. Both YabT and SpoIIE were highly connected, thus emerging like hubs in this network. Particularly, YabT made contact with TkmA and PtkB, suggesting the possibility of interference between the serine/threonine and the tyrosine phosphorylation networks. This assumption was strengthened by two outcomes from our third and last rounds of screenings. We observed that YvcJ, a P-loop containing GTPase with identified kinase activity (Pompeo et al., 2011), interacted with TkmB. Furthermore, we identified several connections between the stressosome controlling Ser/Thr protein kinase RsbT (Kang et al., 1998) and kinase and phosphatase proteins from both the Tyr (PtkA, PtpZ) and Ser/Thr (PrkD) phosphorylation pathways (Figure S1).

**Figure 1:**
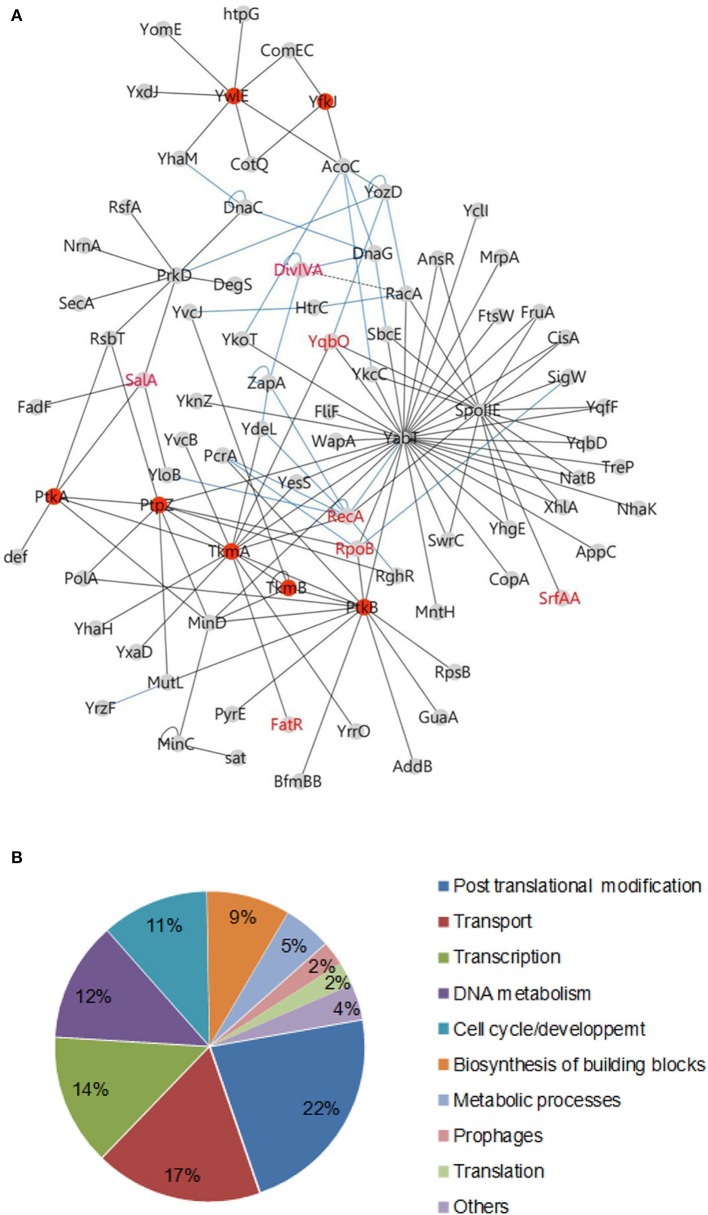
**Protein tyrosine kinases and phosphatases interaction network in *B. subtilis***. **(A)** Binary interactions: The network of protein-protein interaction was illustrated using the software Cytoscape 3.0.1. Protein interacting pairs are connected by full lines. Interactions determined in other studies are illustrated by blue lines (Marchadier et al., 2011) or dashed lines (Wu and Errington, 2003; Jers et al., 2011). Reciprocal interactions have been omitted for more clarity (see Table S1). Members of the tyrosine phosphorylation pathway in *B. subtilis* are indicated as red nodes. Phosphoproteins characterized in the literature are labeled in red. **(B)** Pie chart diagram illustrating the functional classification of interacting proteins according to the biological processes as shown in Table S4.

After addition of connecting edges from the *B. subtilis* interactome (Marchadier et al., 2011), the resulting interaction map of proteins involved in phosphorylation pathways comprises 137 specific interactions linking 82 proteins (Figure 1A, Tables S1, S2). This network is of high confidence and contains many proteins with already documented phosphosites. These positive controls include recently characterized phosphoproteins RecA, phophorylated by YabT (Bidnenko et al., 2013), and FatR, phosphorylated by PtkA (Derouiche et al., 2013). SrfAA and YqbO, which were detected in a previous phosphoproteome study (Macek et al., 2007), also appear in the network connected with YabT and SpoIIE, respectively. DegS, which was previously characterized as a substrate for both YabT and PrkD, was added to the network (Jers et al., 2011). About 44% of proteins were detected as overlapping fragments, delineating discrete minimal interacting domains (Table S2). These high-confidence interactions provide an important validation of our approach. A significant fraction (20%) of the prey proteins were found to interact with both a kinase and a cognate phosphatase (Table 1), suggesting they could be regulated by reversible phosphorylation. These proteins have been grouped into functional categories (Figure 1B, Table S3). Two thirds of them belong to four main functional classes: post translational modifications, transport of metabolites (ions or peptides), transcription and DNA metabolism.

**Table 1:** **Proteins contacted by a kinase and a cognate phosphatase in the *B. subtilis* PPi network**.

**Proteins**	**Function**	**Kinase**	**Phosphatase**
MutL	DNA mismatch repair factor	PtkB	PtpZ
PolA	DNA polymerase I	PtkB	PtpZ
RpoB	RNA polymerase β subunit	PtkB	PtpZ
MinD	ATPase activator of MinC	PtkA/PtkB	PtpZ
RsbT	Ser/Thr Kinase	PtkA	PtpZ
YabT	Ser/Thr Kinase	PtkB	PtpZ
RecA	SOS repair factor/DNA processing	YabT	SpoIIE
RacA	chromosome-anchoring protein	YabT	SpoIIE
DegS	PTS sensory kinase	YabT	SpoIIE
SbcE	DSB repair	YabT	SpoIIE
SwrC	transporter/ swarming	YabT	SpoIIE
YqfF	phosphodiesterase	YabT	SpoIIE
YkcC	putative glycosyltransferase	YabT	SpoIIE
YqbD	putative DNA wielding protein/skin element	YabT	SpoIIE
YqbO	putative lytic transglycosylase/skin element	YabT	SpoIIE
FruA	phosphotransferase system (PTS) fructose-specific	YabT	SpoIIE
CisA	site-specific DNA recombinase	YabT	SpoIIE
XhlA	cell lysis/PBSX	YabT	SpoIIE
YhgE	putative methyl accepting protein	YabT	SpoIIE
NatB	Na+ exporter	YabT	SpoIIE

### New substrates of BY-kinases involved in transcriptional control and DNA metabolism

**Figure 2:**
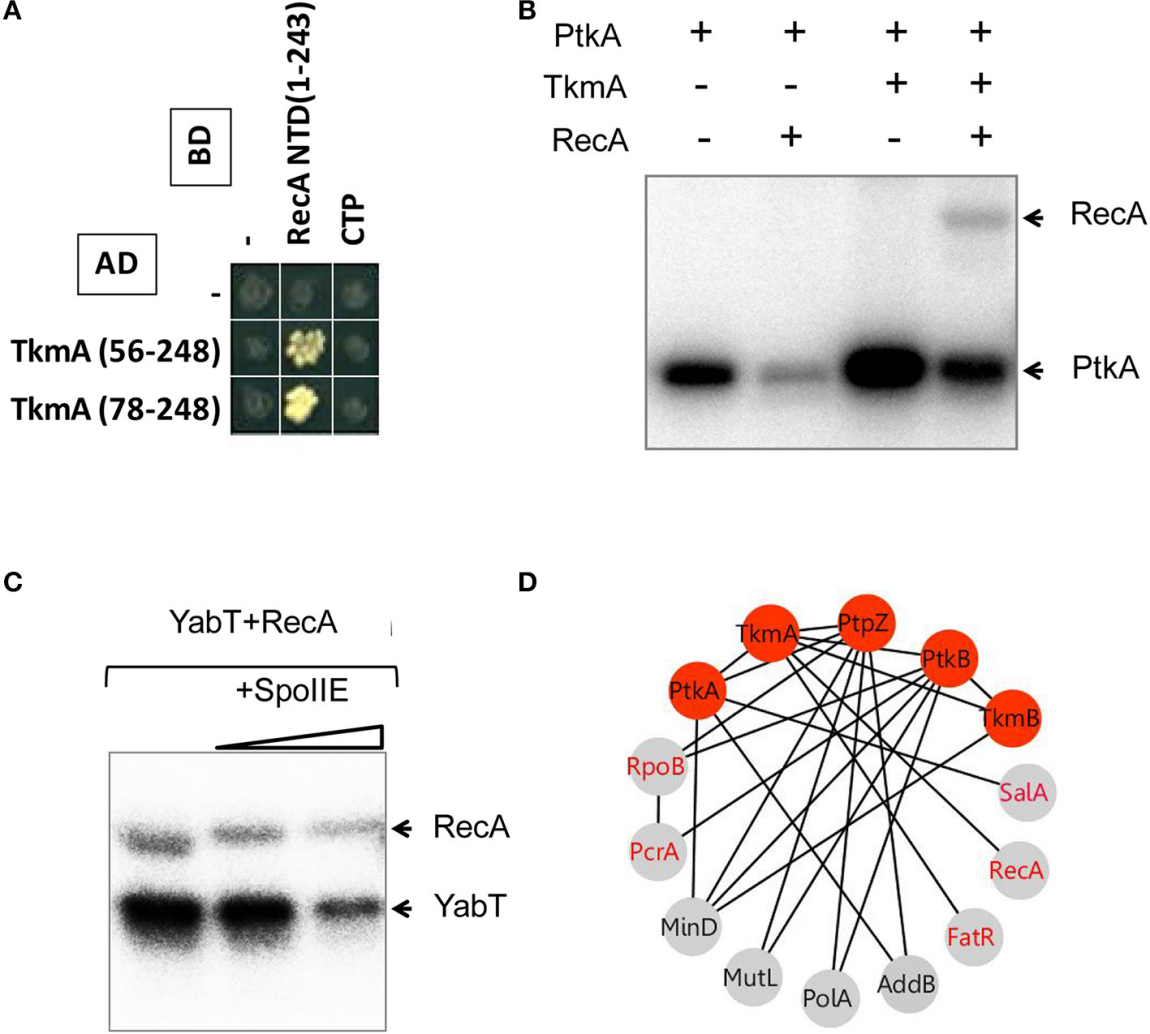
**Phosphorylation of the recombinase protein RecA. (A)** Protein-protein interaction between TkmA and the N-terminal domain (NTD) of RecA assayed by yeast two-hybrid (see experimental procedures). AD and BD refer to activating and DNA binding domain of Gal4 fused to TkmA and RecA, respectively. Interacting domains of TkmA and RecA are indicated. **(B)** *In vitro* phosphorylation of RecA by PtkA in the presence of TkmA. Experiments were performed as described in the experimental procedures. The presence or absence of RecA is indicated as ± above each lane. Bands corresponding to autophosphorylation of kinases and phosphorylated RecA are indicated by arrows. **(C)** *In vitro* phosphorylation and dephosphorylation of RecA mediated by YabT and SpoIIE. Phosphorylation was performed by mixing RecA and YabT as described in the experimental procedures. Note that SpoIIE-mediated dephosphorylation of both RecA (YabT-mediated phosphorylation) and YabT (autophosphorylation) is observed. **(D)** Protein-protein interactions between tyrosine sphosphorylation and DNA-binding proteins. Proteins are indicated by nodes and interactions by edges. Red nodes indicate the components of protein tyrosine phosphorylation/dephosphorylation machinery. Gray nodes indicate DNA-binding proteins. Identified phosphoproteins are labeled in red.

**Figure 3:**
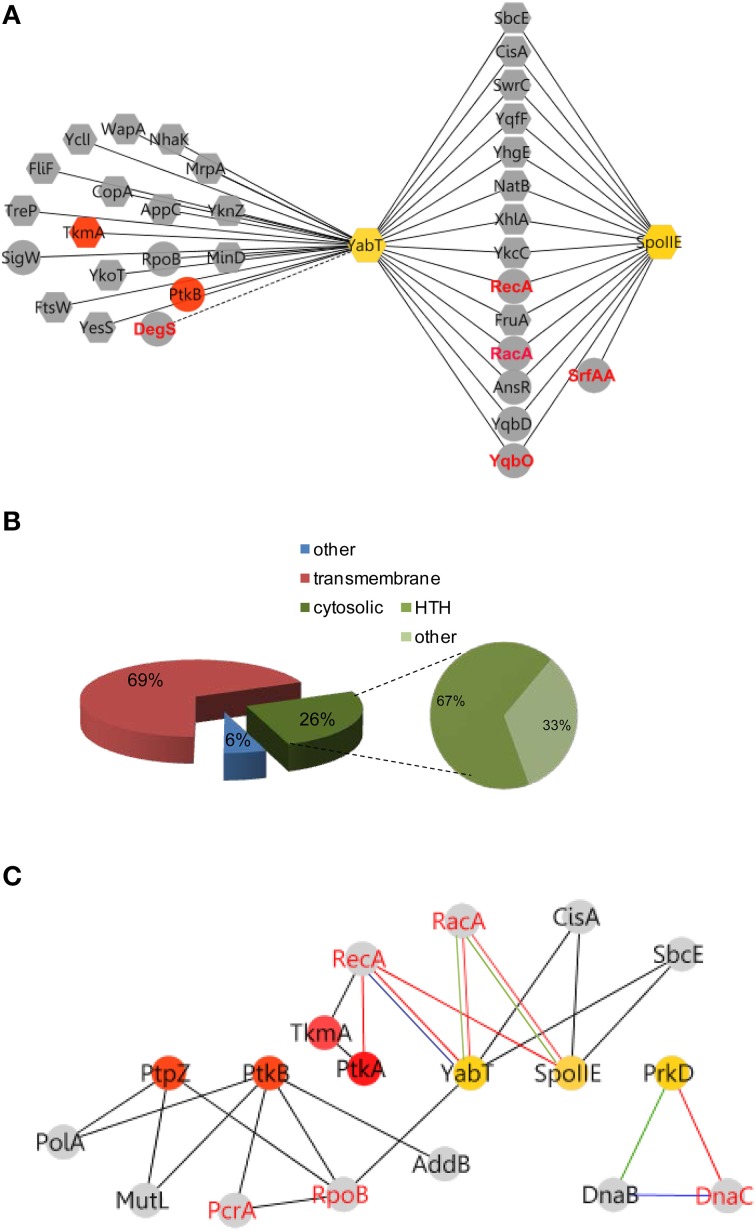
**YabT/SpoIIE centered interacting network highlight membrane protein substrates**. **(A)** Membrane spanning and cytosolic proteins are symbolized by octagonal and round shapes, respectively. Phosphoproteins are labeled in red. **(B)** Pie-Chart illustrating the repartition of protein substrates in the membrane and cytosol. HTH-containing proteins among the cytosolic proteins are represented. **(C)** Cross talk between Ser/Thr/Tyr kinases and pathways of DNA metabolism. Interactions between kinases and partners are indicated by nodes connected with edges. Nodes color refers to Tyr-family (red) and Ser/Thr (yellow) family of kinase. PPI are symbolized by black edges; red edges correspond to *in vitro* phosphorylation validations; *blue* edges indicate *in vitro/in vivo* interactions; green edges symbolize high correlation of expressions (as described in Nicolas et al., 2012). Red node labels indicate phosphoproteins.

In the first set of screens, the network revealed the interaction of PtkA-activator TkmA with a transcription regulator FatR. In a separate study we reported that FatR is phosphorylated by PtkA in the presence of TkmA, at residue Y45 located in the DNA binding domain (Derouiche et al., 2013). The consequence of FatR phosphorylation is its dissociation from its DNA operator sequence with derepression of the *fatR-cyp102A3* operon. The MRP-like gene regulator SalA which was found to interact with PtkA was also proved to be a substrate for phosphorylation (Derouiche A. and Mijakovic I. unpublished result). In this case PtkA-dependent phosphorylation stimulated DNA-binding of SalA, which in turn repressed the transition state regulator *scoC* (Derouiche A. and Mijakovic I. unpublished result). Furthermore, we found that TkmA interacted with the N-terminal domain of the general DNA recombinase RecA (Figure 2A). Supporting this observation, we show here that RecA can be phosphorylated by PtkA in the presence of TkmA *in vitro* (Figure 2B). To date, no direct substrates of the putative BY-kinase PtkB have been identified in *B. subtilis*, since solubility issues precluded *in vitro* studies with PtkB (Mijakovic et al., 2003). Our network revealed several interaction partners of PtkB involved in various processes involving the DNA metabolism (Figures 2D, 3C). These include the RNA polymerase β-subunit RpoB, the DNA helicase PcrA, the DNA polymerase PolA, the ATP-dependent DNA helicase/nuclease AddB and the DNA mismatch repair protein MutL. In fact, with the exception of AddB, these proteins also interact with the phosphotyrosine-protein phosphatase PtpZ, highlighting that they indeed could be targeted for modifications through phosphorylation/dephosphorylation on tyrosine residues. RpoB was previously identified as phosphorylated on tyrosine (Ge et al., 2011) and serine (Sun et al., 2010) residues in *Helicobacter pylori* and *Streptococcus pneumoniae*, respectively. Both RpoB and the UvrD-like helicase PcrA were also reported to be phosphorylated on tyrosine in *Klebsiella pneumoniae* (Lin et al., 2009). In our network, PtkB was also found to interact not only with its cognate modulator TkmB but also with TkmA, the cognate modulator of PtkA, suggesting a cross-talk between the two tyrosine kinase systems. In conclusion, our PPI network revealed a number of new substrates of PtkA which we have experimentally validated. In addition it pointed to potential substrates of PtkB.

### New substrates of the hanks-type Ser/Thr kinases and phosphatases

**Figure 4:**
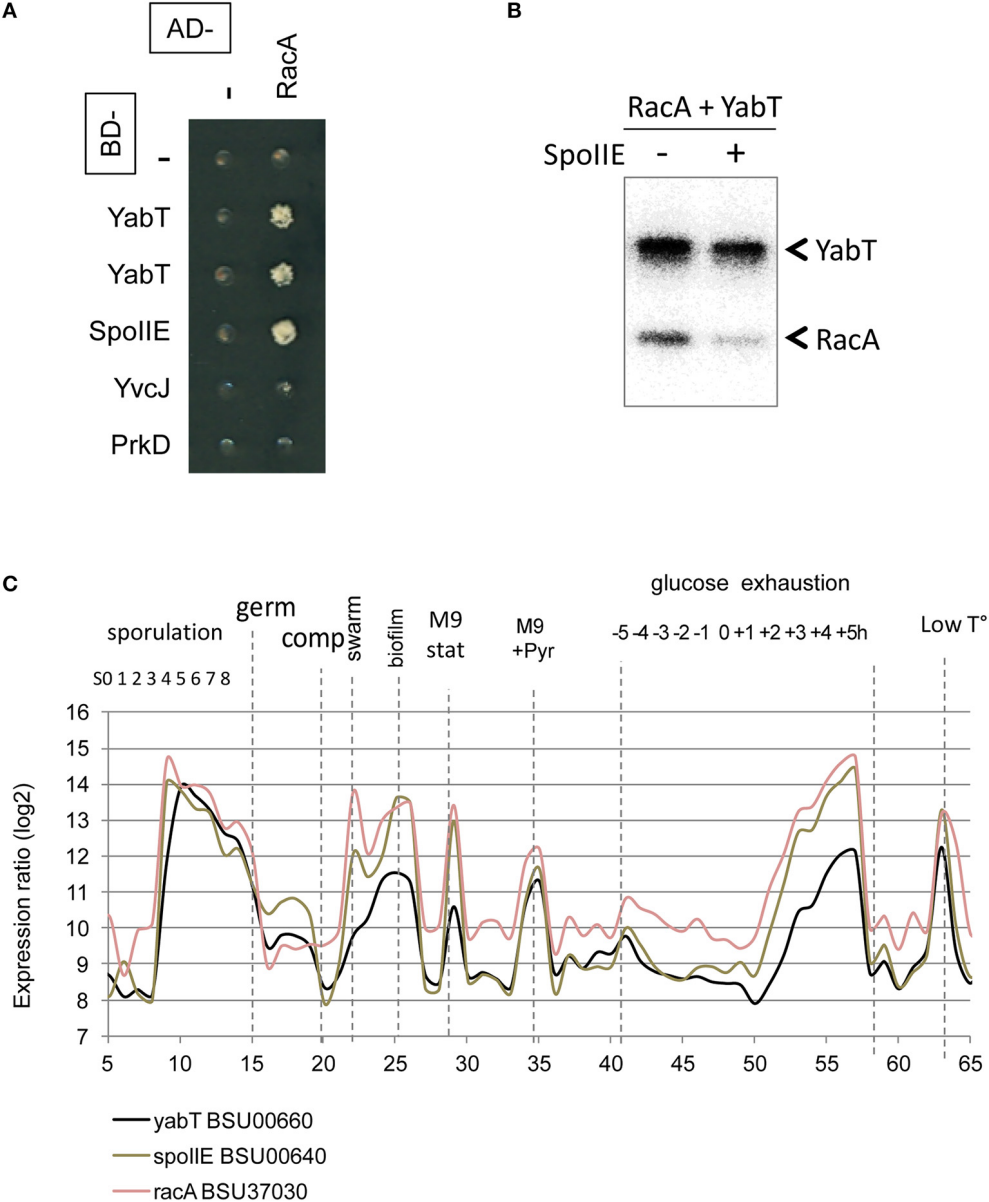
**Reversible Ser/Thr phosphorylation of RacA**. **(A)** RacA interaction with YabT and SpoIIE by yeast 2HB. Gal4 BD- (line) and AD-fusions (column) expressed in yeast haploid cells of complementary mating type were mated and assayed for expression of interaction phenotype as described in the experimental procedures. Diploids co-expressing an AD-RacA fusion (aa 81-194/194) and BD-YabT or BD-SpoIIE are able to grow onto –LUH selective media. **(B)** *In vitro* phosphorylation/dephosphorylation of RacA by YabT, and SpoIIE. Phosphorylation was performed as described in the experimental procedures. **(C)** Condition-dependent transcriptomes of *yabT* (black), *spoII*E (gray) and *racA* (light red), as obtained in Nicolas et al. (2012).

The interaction of the activator TkmA and the Ser/Thr-kinase YabT established in the first round of screens suggested that the tyrosine and serine/threonine phosphorylation pathways could be connected. This connection was further substantiated in the second round, via the Ser/Thr kinase PrkD, the GTPase YvcJ, the RNA polymease β-subunit RpoB, the recombinase RecA, and the transcriptional factors YesS, and YdeL which were found to connect with both BY-kinase and Ser/Thr-kinase nodes. We explored the Ser/Thr-kinase nodes with the same approach to examine whether PPI network allows identification of new substrates. YabT and its cognate phosphatase SpoIIE are encoded by the same operon and are are strongly up-regulated during sporulation. SpoIIE is known to modulate the phosphorylation state of the anti-anti sigma F factor SpoIIAA (Arigoni et al., 1996). YabT is an unusual Hanks-type kinase possessing a transmembrane helix, but no extracellular signal-binding domain. In a recent study we showed that YabT localizes at the septum during spore formation and is activated by binding ssDNA (Bidnenko et al., 2013). Activated YabT phosphorylates the recombinase RecA at the onset of sporulation and regulates its role in maintaining chromosome integrity. Interaction profiles of YabT and SpoIIE revealed numerous protein partners. Indeed, YabT appears as a hub protein that connects 32 proteins, about half of which (including RecA) also interact with SpoIIE (Figure 3A, Table 2). We speculated that proteins interacting with both a kinase and a phosphatase are very likely to undergo phosphorylation. Supporting this hypothesis, we found that RecA, a known substrate of YabT, can be dephosphorylated by SpoIIE (Figure 2C). The DNA-binding developmental protein RacA was also investigated (Figure 4). During sporulation RacA anchors the segregating chromosome at the cell pole in a DivIVA-dependent manner (Ben-Yehuda et al., 2003). Examination of the condition-dependent transcription profiles of *racA* and *spoIIE* shows they are members of the same synexpression group mainly up-regulated during sporulation, indicating that they are involved the same biological process (Figure 4C, Nicolas et al., 2012). The phosphorylation and dephosphorylation of RacA by YabT and SpoIIE, respectively, were confirmed *in vitro* (Figure 4B). This observation highlights a potential role of the kinase YabT in the regulation of RacA activity, and further validates our assumption that interaction partners of a kinase and a phosphatase are presumably substrates for both enzymes.

**Table 2:** **Proteins contacted by more than one kinase in the *B. subtilis* PPi network**.

**Proteins**	**Function**	**Phosphorylation pathways**	
		**Y- kinases**	**S/T-kinases**
MutL	DNA mismatch repair factor	PtkB	YrzF
RpoB	RNA polymerase β subunit	PtkB	YabT
SalA	transcriptional regulator (MarR family)	PtkA	PrkD
MinD	ATPase activator of MinC	PtkA/PtkB	YabT
DegS	Two component histidine kinase		PrkD/YabT
RsbT	Ser/Thr kinase	PtkA	PrkD
YesS	transcriptional regulator (AraC/XylS family)	TkmA	YabT
RecA	SOS repair factor/DNA processing	TkmA/PtkA	YabT/SpoIIE
TkmA	tyrosine kinase PtkA modulator	TkmB/PtkA	YabT
PtkB	tyrosine kinase	TkmA/TkmB	YabT

Classification of all proteins of the YabT hub revealed that 69% of partners are transmembrane proteins, with a high proportion of metabolite- and ion-transporters. Among cytosolic interacting proteins, 67% exhibited HTH motifs suggesting DNA binding properties (Figure 3B). Of note, this parallels the properties of YabT itself, a transmembrane kinase activated by DNA binding (Bidnenko et al., 2013). In addition to RecA and RacA, YabT/SpoIIE were found to interact with SbcE, a structural maintenance of chromosome (SMC)-like protein involved in DNA double-strand break repair (Krishnamurthy et al., 2010) and CisA, a site-specific DNA recombinase necessary for reconstitution of the sigma factor K during sporulation (Kunkel et al., 1990) (Figure 3C). Given the ability of YabT to be stimulated by ssDNA, our identification of potential substrates with DNA-binding properties and/or proteins involved in DNA metabolism substantiates its observed involvement in maintenance of DNA integrity (Bidnenko et al., 2013).

**Figure 5:**
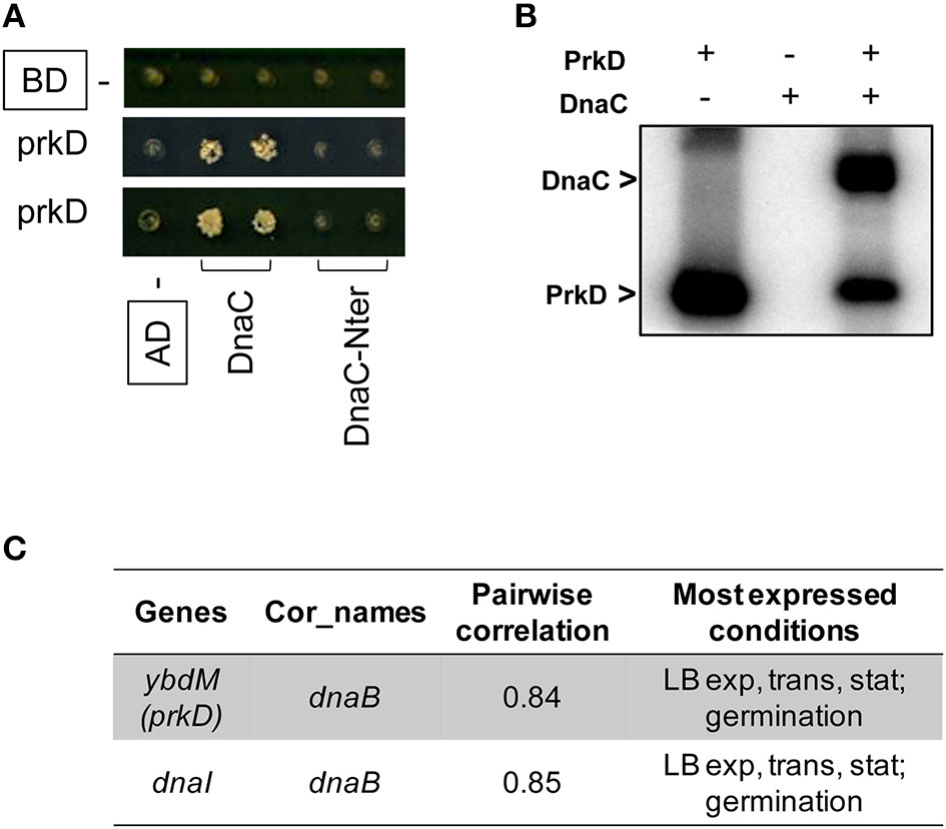
**Phosphorylation of the DNA helicase DnaC. (A)** Protein-protein interaction between PrkD and DnaC assayed by yeast two hybrid. AD and BD refer to activating and DNA binding domain of Gal4 fused to PrkD and DnaC, respectively. DnaC-Nter refers to the N-terminal domain of dnaC, spanning residues aa 1–170. **(B)** *In vitro* phosphorylation of DnaC by PrkD. Phosphorylation was performed as described in experimental procedures. Bands corresponding to autophosphorylation of PkrD and phosphorylated DnaC are indicated by arrows. **(C)** Correlations between transcriptional levels of *prkD* and the components of the DNA helicase loader complex. Pairwise correlations between DnaC, DnaB, and DnaI values were calculated over the 104 physiological conditions (Nicolas et al., 2012).

Further, our PPI network revealed a direct interaction between the YabT paralogue PrkD and the replicative DNA helicase DnaC (Figure 5, Tables S1, S2). Interaction of the kinase with DnaC in yeast two-hybrid requires the DnaC C-terminal domain comprising the helicase function. We confirmed the ability of PrkD to phosphorylate DnaC *in vitro* (Figure 5B), suggesting that PrkD could be involved in regulating DnaC activity. DnaC loading onto a melted single strand DNA fork is assisted by DnaB and DnaI (56). Analysis of condition-dependent transcriptome of *B. subtilis* shows that *prkD* (old name *ybdM*) and *dnaB* exhibit a high pairwise correlation throughout ~100 different physiological conditions (Nicolas et al., 2012; Figure 5C), further strengthening the notion that PrkD could participate to the regulation of chromosomal replication. In conclusion, our PPI network revealed that both YabT and PrkD interact with, and phosphorylate, proteins with crucial roles in DNA metabolism.

### MinD, a new modulator of the BY-kinase PtkA

**Figure 6:**
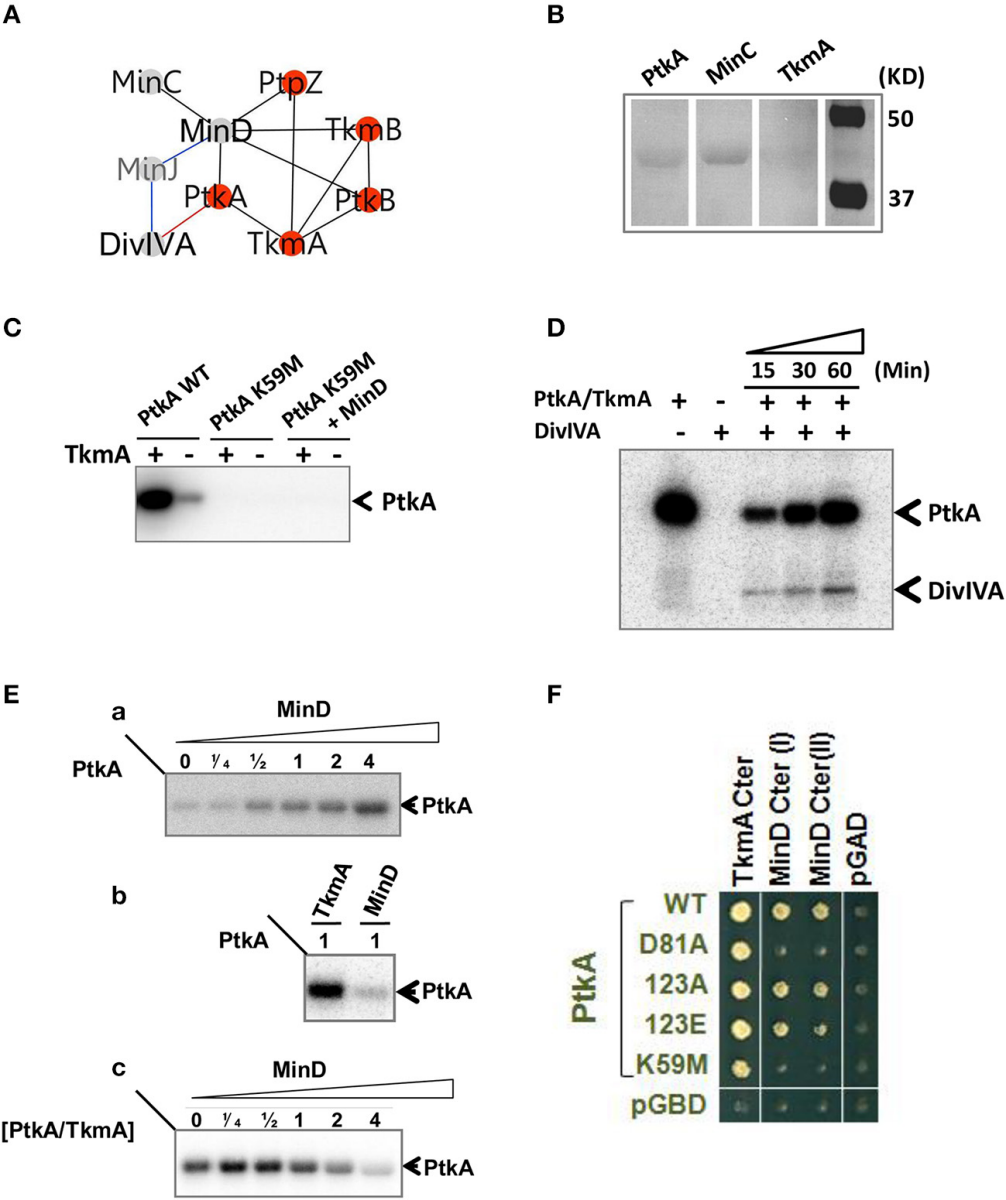
**Functional analysis of MinD/PtkA interaction**. **(A)** MinD-centered interaction networks. Interactions are illustrated by edges connecting nodes (proteins). Interactions were identified by yeast 2HB (black), genetic (blue) and *in vitro* (red). **(B)** Interaction between PtkA and MinD detected by far-western blotting. PtkA, MinC, and TkmA were separated by SDS-PAGE and transferred onto nitrocellulose membrane. Interaction was determined by strep tagged MinD which was revealed by Strep-Tactin. MinC acts as a positive control and TkmA acts as a negative control. **(C)** MinD is not acting as a kinase. All proteins were mixed in equimolar concentration. The presence or absence of TkmA is indicated as ± above each lane. Bands corresponding to phosphorylated PtkA are indicated by arrows. **(D)** *In vitro* phosphorylation of DivIVA by PtkA in the presence of TkmA. The presence or absence of proteins is indicated as ± above each lane. Time dependence of the reaction was followed and the incubation times (15, 30, or60 mins) are given above the lanes. Bands corresponding to autophosphorylated PtkA and phosphorylated DivIVA are indicated by arrows. **(E)** MinD acts as an activator of PtkA. Autoradiography of SDS-polyacrylamide gels showing the influence of MinD on autophosphorylation of PtkA. **(a)** PtkA was incubated with increasing concentrations of MinD. The ratio of MinD to PtkA was indicated above each lane. **(b)** MinD activates PtkA less efficiently than TkmA. PtkA was incubated with equivalent concentration of TkmA or MinD. **(c)** MinD can compete with TkmA for PtkA activation. TkmA/PtkA were incubated in the presence of increasing amount of MinD. The ratio of MinD to TkmA/PtkA was indicated above each lane. Bands corresponding to autophosphorylated PtkA are indicated by arrows. **(F)** Yeast two-hybrid interaction between MinD and PtkA. C-terminal domain of TkmA (aa187–248) and C-terminal domains of MinD (I: aa135–268; II: aa89–268) were fused to the activating domain of Gal4 (AD, labeled in black). PtkA wt and mutant derivatives (as indicated) are fused with the binding domain of Gal4 (BD, labeled in green). PGAD and pGBDU are control empty plasmids expressing the Gal4-AD and BD domains.

The MinD ATPase, required for the correct positioning of the division site, was identified as interacting partner of the two BY-kinases, PtkA and PtkB, as well as the phosphotyrosine-protein phosphatase PtpZ (Figure 6A). This pointed to MinD as a potential target for tyrosine phosphorylation. However, we found that MinD was not phosphorylated by PtkA *in vitro* (data not shown). We first validated the interaction between MinD and PtkA by far Western blotting (Figure 6B). In this assay we detected the expected MinD-MinC complex and confirmed the physical interaction between MinD and PtkA, but not TkmA, in agreement with the two-hybrid. MinD is an ATPase from the P-loop NTPase superfamily previously reported to be a close structural homolog of BY-kinases, but lacking the C-terminal tyrosine cluster necessary for kinase autophosphorylation (Mijakovic et al., 2005b). We therefore explored potential kinase activity of MinD *in vitro* (Figure 6C). MinD was found unable to autophosphorylate or to phosphorylate a PtkA derivative carrying a substituted catalytic residue (K59M), corroborating that MinD does not act as a kinase. During cytokinesis, MinD is known to functionally interact with the cell division initiator protein DivIVA in the presence of the bridging protein MinJ, by preventing the formation of a septum near the cell pole (Levin et al., 1992; Lee and Price, 1993; Marston et al., 1998). Interestingly, we found that DivIVA could undergo phosphorylation by PtkA in the presence of TkmA (Figure 6D).

We then tested the ability of MinD to promote the activation of PtkA in the absence of TkmA *in vitro* (Figure 6E). Incubation of PtkA with increasing amounts of MinD activated its autophosphorylation, indicating that MinD is able to act as modulator of PtkA activity similar to TkmA (Figure 6Ea). However, incubation of PtkA with TkmA or MinD revealed that MinD-mediated PtkA activation was much less efficient compared to TkmA (Figure 6Eb). Finally, we showed that in the presence of excess of MinD, the activation of PtkA in the presence of TkmA was inhibited, indicating that MinD was able to compete with TkmA for binding to PtkA (Figure 6Ec). We investigated the functional elements of PtkA required for interaction with MinD and TkmA by looking for their ability to interact with several PtkA mutant derivatives in yeast two-hybrid (Figure 6F). The mutants included substitutions of key active site residues (catalytic K59M and Mg-coordinating D81A) and the tyrosine autophosphorylation sites (Y225, Y227, and Y228 changed into F). Mutations within the C-terminal tyrosine cluster did not affect the PtkA interaction with either TkmA or MinD. Interestingly, the substitution of either active site residues specifically impaired PtkA interaction with MinD but not with TkmA. This result points to different modes of binding of TkmA and MinD to PtkA.

### PtkA plays a role in cell division

**Figure 7:**
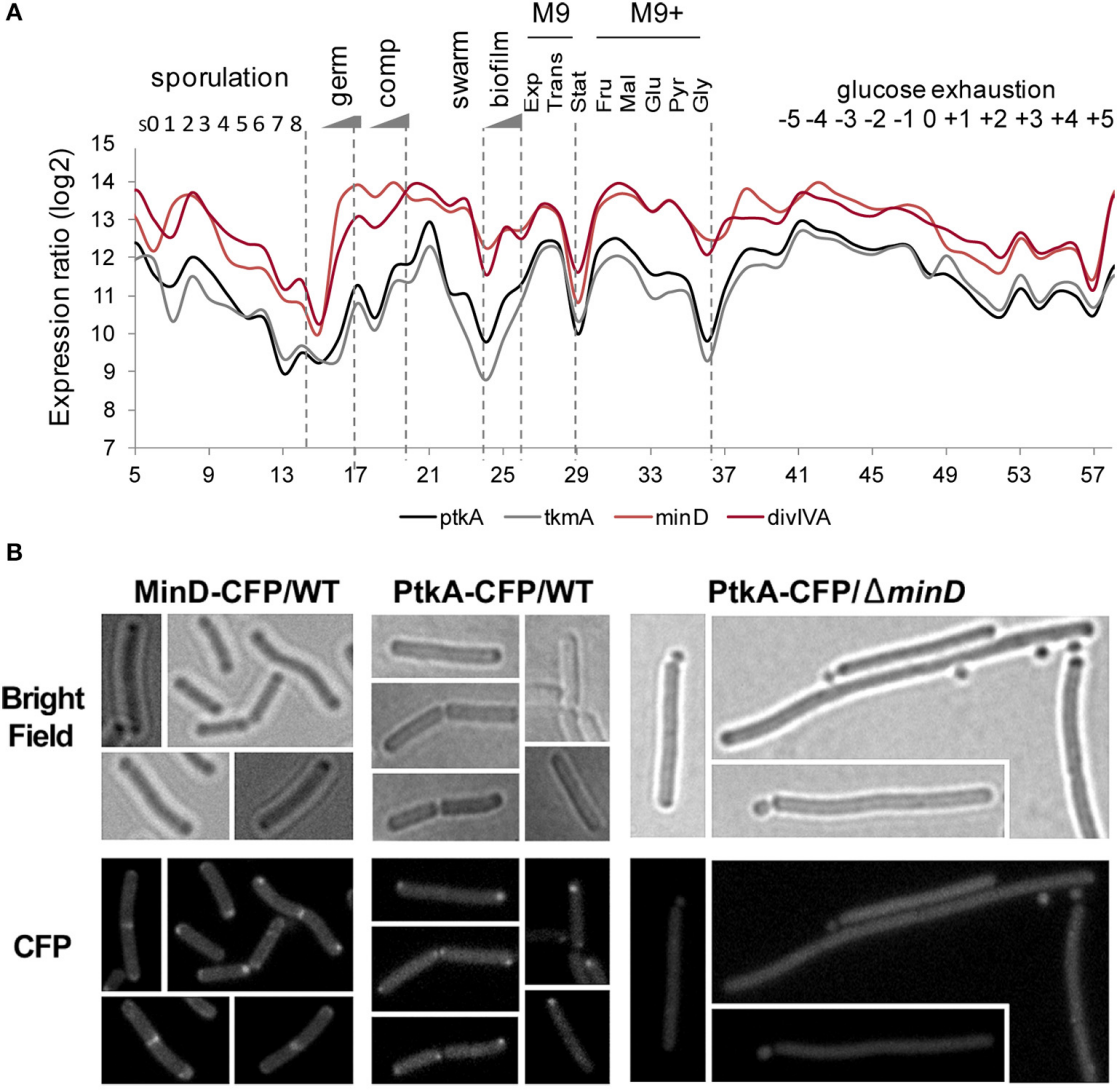
**PtkA/MinD cellular expression and localization**. **(A)** Condition-dependent transcription profiles of *minD, divIVA, ptkA, and tkmA* (Nicolas et al., 2012). PtkA and TkmA expression profiles are in black and gray, respectively. Expressions of DivIVA and MinD are in red (dark and light, respectively). **(B)** MinD-dependent localization of PtkA: GFP fluorescence (upper panel) and bright field (lower panel) images are shown. Localization of MinD in wild type cells (left panel) and localization of PtkA in wild type (middle panel)and in Δ*minD B. subtilis* cells (right panel) as indicated. Arrows point to minicells.

**Figure 8:**
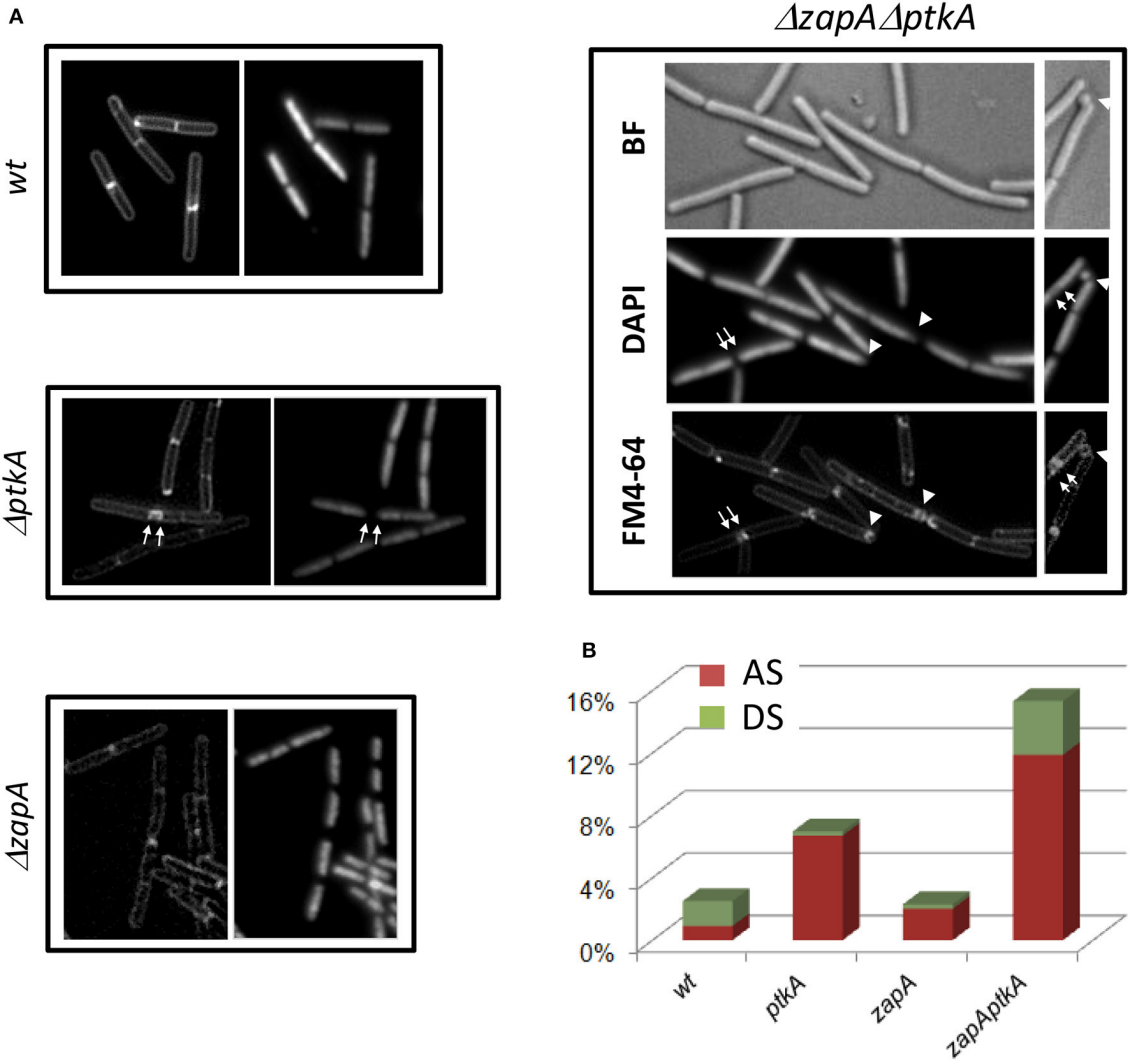
**PtkA is involed in regulation of correct septal positioning**. **(A)** Δ*ptkA* cells in wild type or *zapA* deficient background were observed by fluorescent microscopy. Cells were visualized by membrane staining (FM4-64, or left panel), nucleoid staining (Dapi, or right panel), or bright field (BF). Septum defects are indicated by white arrows (double septum) and white triangles (asymmetric septum or anucleated cell). **(B)** Defaults in septal formation in each strain are estimated as % over a total number of cells >500. AS: asymmetric septum, red; DS: double septum, green.

Transcription profiles of *minD*, *divIVA*, *ptkA*, and *tkmA* exhibit a high degree of correlation throughout all physiological conditions, suggesting they function in the same biological process (Figure 7A). A close examination of *ptkA* single mutant revealed a mild cell division defect with a noticeable proportion of cells containing double septa (7%), compare to the wild type cells (<1%) (Figures 8A,B). We investigated for PtkA-associated phenotypes in cells deficient in the division factor *zapA*. *B. subtilis zapA* mutant exhibits no remarkable division phenotype (Gueiros-Filho and Losick, 2002). However, a *zapA* null mutation was described to exacerbate cytokinesis block-induced phenotypes when combined with other actors of cell division (Gueiros-Filho and Losick, 2002; Dempwolff et al., 2012; Surdova et al., 2013). In rich media, *zapA ptkA* double mutant exhibited a higher proportion of cell with double septa (12%) as well as cells containing asymmetric septa (3%), sometimes leading to anucleated cells (Figures 8A,B). These observations suggest a role of PtkA in the regulation of cytokinesis.

To further understand the role of MinD in PtkA activity, we examined their subcellular localization. MinD is known to exhibit a very specific localization pattern at the cell poles as well as at mid cell. When fused to CFP, PtkA was found to localize as a single focus, preferentially at the old cell poles of elongating cells (Figure 7B). The absence of MinD typically leads to the formation of anucleated minicells, resulting from the mislocalisation of septum at the cell poles (Burmann et al., 2011). Here we found that the inactivation of *minD* also abolished the polar localization of PtkA foci, indicating it is MinD-dependant. This result provides *in vivo* validation of the MinD/PtkA interaction and hints at a role of MinD in holding PtkA at the cell pole during cytokinesis.

## Discussion

A hallmark of BY-kinases from Gram-positive bacteria is that the cytosolic kinase and its transmembrane activator are individual proteins encoded by separate adjacent genes. This differs from their Gram-negative counterparts where the two activities are encoded by the same gene. A functional consequence of this particular architectural feature could be that it enables the kinases from Gram-positive bacteria to interact with alternative activators, which may provide different substrate specificities. We presented here a protein-protein interaction landscape of the tyrosine phosphorylation network of *B. subtilis*. Combined with a systematic and experimental elimination of false positives, our approach has proved effective for identifying numerous substrates of BY-kinases and phosphatases, as well as new proteins that modulate their activity. This PPi network not only provided a kinase context for already identified phosphoproteins, but also identified new protein partners for kinases, including other kinases and phosphatases. This network can be considered of high biological relevance, with numerous proteins being contacted by both a kinase and cognate phosphatase, strongly suggesting they are true substrates or they belong to the same regulatory assembly. Our examination of kinase/substrates and kinase/modulators from the tyrosine but also the serine/threonine phosphorylation pathways corroborated these assumptions. This study highlights the role of protein phosphorylation in the regulation of various aspects of the *B. subtilis* cell cycle as DNA replication, transcription, cell division and sporulation.

Common signaling mechanisms often involve multisite protein phosphorylation to regulate the various activities of a protein whether carried out by the same kinase or by the sequential action of several kinases. In *B. subtilis*, the best characterized example is the two-component histidine kinase DegS, which was shown to be phosphorylated by the two Ser/Thr kinases PrkD and YabT *in vitro* (Jers et al., 2011). PrkD-dependent phosphorylation was specific to serine 76 located in the signal sensing domain and led to increased efficiency of phosphotransfer to DegU. *In vivo*, DegS phosphorylation at serine 76 was shown to affect various aspects of cellular behavior such as competence, swarming and motility. Our *B. subtilis* interaction network also revealed that other proteins can be phosphorylated by more than one kinase (Table S3-B). A notable example is the multifunctional DNA recombinase protein RecA recently found regulated by phosphorylation on a serine (Lusetti and Cox, 2002; Butala et al., 2009; Bidnenko et al., 2013). Here, our data suggest that tyrosine phosphorylation could also play a role in the regulation of RecA activity. RecA binding to TkmA involves its N-terminal moiety, which is required for the formation of a presynaptic nucleoprotein filament (Lee and Wang, 2009). Whether this activity could be modulated by tyrosine phosphorylation in the cell remains to be determined. Dual phosphorylation involving modification on tyrosine, serine or threonine could also be proposed for the transcriptional regulators SalA and YesS as well as for the RNA polymerase subunit RpoB, which was found to interact with more than one component of the Ser/Thr and Tyr phosphorylation pathways. Together, these observations suggest the existence of dynamic regulations of protein activities in *B. subtilis* cells, where multiple and sequential phosphorylation events could mediate integration of several input signals to better adjust the cellular response to environmental changes, similar to what is described in eukarya.

Remarkably, our network reveals important crosstalk among different classes of kinases. Interactions were observed between components of the Tyrosine and Ser/Thr phosphorylation pathways, as well as among Ser/Thr-kinases. These connections strongly suggest that kinases might phosphorylate each other, generating signaling cascades similar to what was observed in eukarya. This hypothesis has been explored in a separate study, in which the ability of all soluble *B. subtilis* BY- and Ser/Thr kinases to phosphorylate each other *in vitro* was explored. This assay revealed many trans-phosphorylations at functionally critical residues indicative of kinase regulation by phosphorylation (Shi et al., 2014). A similar study carried out recently in *M. tuberculosis* also provided strong evidence for a hierarchical and spatially organized crosstalk among Ser/Thr kinases in this bacteria (Baer et al., 2014). These results support the existence of kinase cross-phosphorylation to form complex eukaryotic-like signaling networks in bacteria. Furthermore, promiscuous binding of BY-kinases PtkA and PtkB with different modulators was observed throughout the network suggesting a functional interplay. Whether PtkB performs *in vivo* as a kinase remains to be determined. An alternative hypothesis could be that PtkB acts as a pseudokinase to mediate interaction with protein substrates for subsequent tyrosine modification by PtkA. Our data also hints at a potential a role of modulators in recruiting some substrates. In agreement with this notion is the finding that the transcriptional regulator FatR, which interacts with TkmA, is regulated by PtkA (Derouiche et al., 2013). On the whole, our PPi network highlights elaborate mechanisms for phosphorylation of tyrosine in the signal transduction pathways that regulate various aspects of DNA metabolism.

A remarkable feature of this PPi network is the central position of the cell division regulator protein MinD which appears as a hub protein densely connected with many members of the tyrosine phosphorylation pathways. MinD shares sequence and structural homology with BY-kinases, but lacks crucial tyrosine residues involved in phosphoryltransfer activity (Mijakovic et al., 2005b). As such, MinD could be considered as a pseudokinase with a non enzymatic-like function. Our resultes indicate a role of MinD in the activation of PtkA *in vitro*, as well as in its *in vivo* localization as a focus at the cell poles. It has been previously reported that PtkA co-localizes with its transmembrane adapter TkmA at the cell membrane in exponentially growing cells (Jers et al., 2010). However, in the previous study *ptkA* and *tkmA* genes were simultaneously overexpressed which may account for this discrepancy. In our present study the localization was detected with wild type expression levels from the *ptkA* natural promoter, without overexpression of *tkmA*. Based on these results, we propose that MinD could act as a platform protein that would tether PtkA at a specific cellular area. Additionally MinD would also act as an activator of PtkA for signal transmission to DivIVA. The biological significance of DivIVA phosphorylation by PtkA during cystokinesis in *B. subtilis* remains to be investigated, but our data clearly points to a role of PtkA in the regulation of cytokinesis. This is in agreement with the cell cycle phenotype of the *ptkA* mutant which was reported previously (Petranovic et al., 2007). A parallel can be drawn between the MinD/PtkA interplay and the A parallel can be drawn with scaffold-directed assembly of signaling components in eukarya. Eukaryotic scaffold proteins are often described as catalytically inactive kinases that can bring together multiple components of signaling cascades, regulating and promoting their interactions at particular localizations in the cell. An example of this process is the Ste5 protein scaffold in the mitogen-activated protein kinase (MAPK) pathway, which directs mating signaling by promoting an activation cascade mediated by three insulated protein kinases MAPKKK Ste11, MAPKK ste7 and MAPK Fus3 (Good et al., 2009). Here, MinD was found connected with other kinases besides PtkA (PktB, YabT) and a phosphatase (PtpZ) thus bringing various components of tyrosine, but also serine/threonine phosphorylation pathways in close proximity. A hypothesis worth exploring would be whether MinD could orchestrate the intracellular location of several kinases and phosphatases, similar to eukaryal anchoring proteins (Pawson and Scott, 1997).

In several bacteria, the phosphorylation of DivIVA was shown to be a key step in the regulation of cytokinesis. So far, this regulation is known to be mediated by Ser/Thr Hank-type kinases. In *Streptomyces coelicolor*, the polarization machinery is regulated by AfsK, a Ser/Thr protein kinase which localizes at hyphal tips and phosphorylates DivIVA on its C-terminus (Hempel et al., 2012; Saalbach et al., 2013). In *S. pneumoniae*, StkP localizes at the division site and specifically phosphorylates DivIVA on threonine 201 (Fleurie et al., 2012). In *Mycoplasma pneumoniae*, phosphorylation of the DivIVA homolog Wag31 by S/T kinases PknA/PknB affects cell shape control (Kang et al., 2005). Our results indicate that in *B. subtilis*, this regulation may involve the phosphorylation of DivIVA on a tyrosine residue.

In conclusion, this study shows that bacterial regulatory network based on protein phosphorylation is considerably more complex and inter-connected than previously believed. The BY-kinase sub-network is inextricably connected to the Ser/Thr-kinase sub-network. Together with the separate study (Shi et al., 2014) providing evidence that kinases can cross-phosphorylate each other, our data highlighted that BY-kinases are activated by alternative modulators, and that substrates are phosphorylated by different kinases, leading to a better integration of a multitude of signals. Significant additional efforts will be required to decipher the functioning of this complex and dynamic regulatory network.

### Conflict of interest statement

The Associate Editor, Christophe Grangeasse, declares that, despite having collaborated with authors Ivan Mijakovic and Lei Shi, the review process was handled objectively and no conflict of interest exists. The authors declare that the research was conducted in the absence of any commercial or financial relationships that could be construed as a potential conflict of interest.
