# Supplementary material for: Protein-tyrosine phosphorylation interaction network in Bacillus subtilis reveals new substrates, kinase activators and kinase cross-talk
Source: Front Microbiol. 2014 Oct 22;5:538. doi: 10.3389/fmicb.2014.00538 (PMC4205851; doi:10.3389/fmicb.2014.00538)
Supplement: Supplementary file 5 [file Presentation1.PDF]

Supplementary Material

Protein-tyrosine phosphorylation interaction network in Bacillus subtilis reveals new substrates, kinase activators and kinase cross-talk.

Lei Shi[1,2], Nathalie Pigonneau[1], Magali Ventroux[1], Abderahmane Derouiche[1,2], Vladimir Bidnenko[1], Ivan Mijakovic[1,2] and Marie-Françoise Noirot-Gros[1*]

[1] INRA, UMR1319 Micalis, Jouy-en-Josas, 78350, France
[2] Systems and Synthetic Biology, Department of Chemical and Biological Engineering, Chalmers University of Technology, 41296 Gothenburg, Sweden

*** Correspondence:** Marie-Françoise Noirot-Gros, INRA, UMR1319 Micalis, Domaine de Vilvert 78350 Jouy-en-Josas, France, Email: marie-francoise.gros@jouy.inra.fr

Supplementary Data

1. Supplementary Figures and Tables

1.1. Supplementary Figures

Supplementary Figure 1.

Figure S1: Yeast two-hybrid screenings of B. subtilis genomic library centered on tyrosine phosphorylation pathway (kinases and phosphatases). In a first round of screenings, the tyrosine kinases PtkA and PtkB, the tyrosine kinase modulators TkmA and TkmB, and the tyrosine phosphatases PtpZ, YwlE and YfkJ were used as baits (red nodes). In the second round of screening some protein prey partners such as the PtkA structural homologues SalA and MinD as well as the serine/threonine kinase YabT and its cognate phosphatase SpoIIE were transferred as baits (blue nodes). The third and final rounds of screenings focused onMinC and YvcJ. Additional interactions were observed by direct mating of preys and baits (framed).

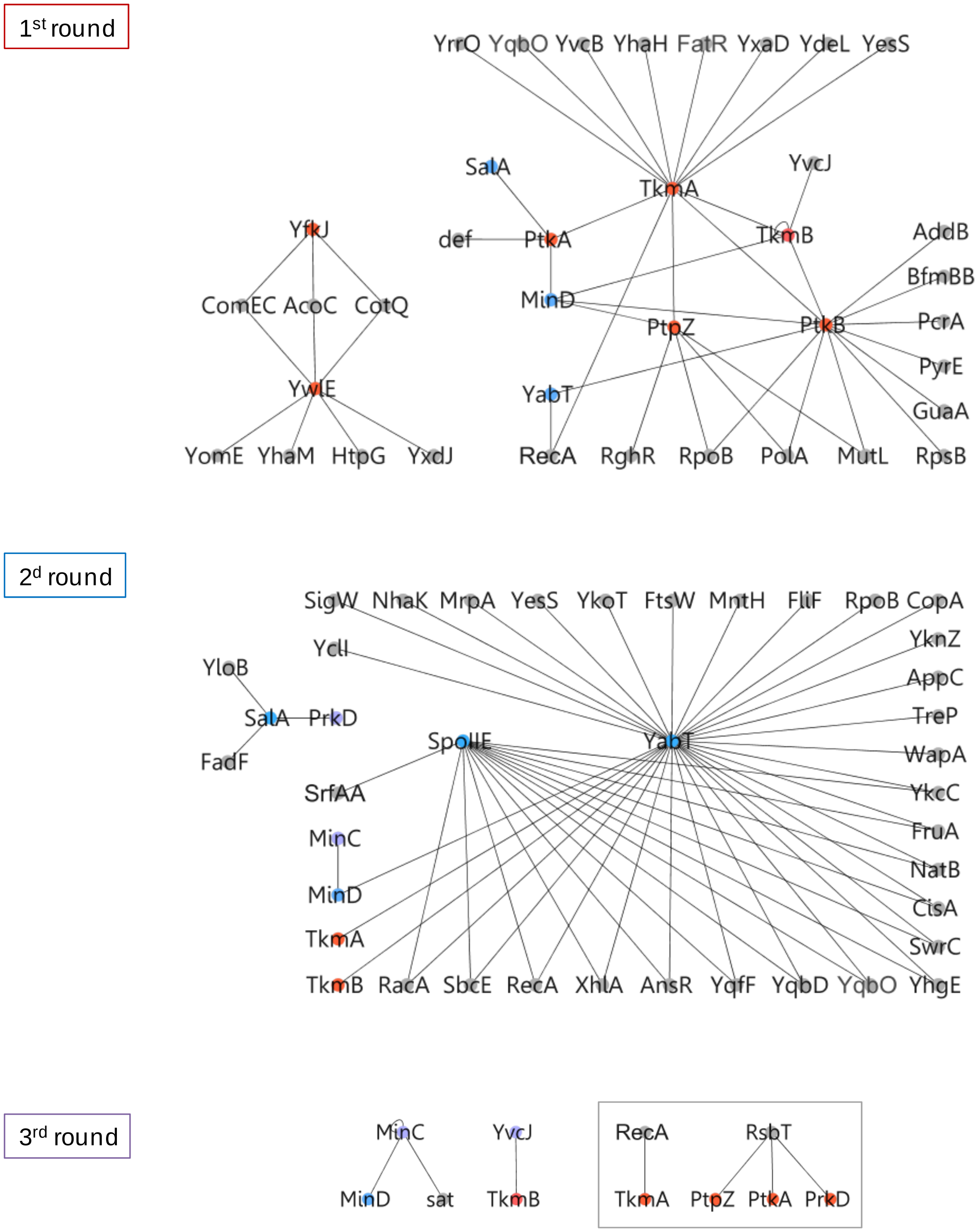

Figure S1: Yeast two-hybrid screenings of *B. subtilis* genomic library

1.2. Supplementary Tables

Supplementary Table 1.

Table S1.xlsx

Table S1: Results from yeast two-hybrid screenings of the *B. subtilis* genomic library and PPi matricial (binary) assays: Genes encoding bait and prey proteins, were listed by gene names. Prey protein function and identified interacting domains are indicated. Class refers to the identification of preys proteins as several overlapping fragments (C1) or unique but specific fragment (C2). The specificity of all interactions were assayed as described in the experimental procedures. The protein interactions have been submitted to the IMEx (http://www.imexconsortium.org) consortium through IntAct (Orchard et al., 2014) and assigned the identifier IM-22270.

Supplementary Table 2.

Table S2.xlsx

Table S2: Summary of interacting pairs within completed PPi network: Bait and Prey interacting pairs with the prey minimal specific interacting domains (SIDs) are indicated. When obtained as a collection of overlapping fragments, prey SIDs correspond to the minimal domain boundaries necessary for interaction. Biological relevance of interactions supported by experimental validations provided in this work or/and in independent studies are mentioned. A given interaction is qualified a highly biologically relevant when i) identified between a bait and several overlapping fragments of a prey or ii) between a prey and two mutated variants of the same bait or iii) when a given prey protein is contacted by both a kinase and its cognate phosphatase (qualified as "potential substrate", see also Table S3A) . These data are also illustrated in the accompanying pie chart.

Supplementary Table 3.

Table S3.xlsx

Table S3: **Functional classification of the protein-protein interaction network centered on Y-kinase in *B. subtilis.*** Functional classes and access to information from the main *B. subtilis* databases are given through web site links to BsubCYC.

Supplementary Table 4.

Table S4.xlsx

Table S4: **List of oligonucleotides used for cloning and strain constructs.**

Table 5: Purification of heterologously expressed proteins. Protein purity was estimated by scanning densitometry of coomassie stained SDS-Page gels.

	Purity(%)
PrkD	70
TkmA	90
MinD	90
MinC	99
$PtkA^{WT}$	95
RecA	99
$PtkA^{K59M}$	40
SpoIIE	75
YabT	90
RacA	99
DnaC/I	99
